# Effect of empagliflozin on human primary cardiomyocytes in a chemically induced hypoxia by CoCl_2_

**DOI:** 10.14814/phy2.70653

**Published:** 2025-12-10

**Authors:** Marek Samec, Michal Pokusa, Andrea Evinova, Ivana Baranova, Eva Baranovicova, Jan Strnadel, Martin Pec, Martin Jozef Pec, Matej Samos, Renata Pecova

**Affiliations:** ^1^ Department of Medical Biology, Jessenius Faculty of Medicine in Martin Comenius University in Bratislava Martin Slovakia; ^2^ Biomedical Centre Martin, Jessenius Faculty of Medicine in Martin Comenius University in Bratislava Martin Slovakia; ^3^ Department of Pathological Physiology, Jessenius Faculty of Medicine in Martin Comenius University in Bratislava Martin Slovakia; ^4^ Biobank for Cancer and Rare Diseases, Jessenius Faculty of Medicine in Martin Comenius University in Bratislava Martin Slovakia; ^5^ Department of Internal Medicine I, Jessenius Faculty of Medicine in Martin Comenius University in Bratislava Martin Slovakia

**Keywords:** cardiomyocyte, empagliflozin, hypoxia, metabolites, miRNA, mitochondria

## Abstract

Cardiovascular diseases (CVD) are the leading cause of premature death and disability. Hypoxic conditions play a central role in the pathophysiology of all CVD. Empagliflozin (EMPA), a sodium‐glucose cotransporter 2 (SGLT2) inhibitor used for diabetes mellitus type II therapy, has demonstrated a beneficial role in improving cardiovascular outcomes for patients with heart failure. Our study aimed to assess the cardioprotective effect of EMPA on primary human cardiomyocytes in a chemically induced hypoxia model. The cardioprotective effect of the SGLT2 inhibitor was evaluated through four individual experiments including: (1) evaluating mitochondrial network integrity, (2) determining cell count, (3) metabolomic profiling, and (4) determining alterations in miRNA expression. After 24 h of EMPA treatment, we observed a significant improvement in mitochondrial network complexity, as evidenced by increased branching (*p* < 0.05) and a reduced number of rod‐shaped mitochondria (*p* < 0.05) in EMPA‐treated cells compared to controls. After cobalt treatment, we didn't observe any protective effect of EMPA in cells affected by cobalt in various biological aspects, including miRNA expression, metabolomics, or viability. Although EMPA treatment was not able to propagate beneficial effects in the presence of cobalt, pretreatment of cells with EMPA indicated a potential cardioprotective effect associated with improving mitochondrial morphology.

## 1 INTRODUCTION

Cardiovascular diseases (CVDs) are currently among the most common causes of mortality worldwide, with incidence and prevalence significantly increasing over the years (Rethemiotaki, 2023). CVDs generally affect various conditions that influence the blood circulation system, including the heart and vessels responsible for transporting and delivering blood (Thiriet, 2019). CVD is divided into several main categories, such as coronary artery disease, cerebrovascular disease, coronary heart disease, peripheral vascular disease, and aortic atherosclerosis (Thiriet, 2019). The heart is a complex organ consisting of a collection of specialized cells, including cardiomyocytes and non‐myocyte cells such as endothelial cells, fibroblasts, and immune system cells (He et al., 2024). Cardiomyocyte dysfunction is closely linked to the development of various CVDs, and its mechanisms can be categorized into ischemic and nonischemic types. Ischemia is known as an essential cause of injury to cardiomyocytes. In this situation, cardiomyocytes do not receive adequate oxygen, other nutrients, and survival factors, causing massive cell death that leads to the development of heart disease and heart failure. Current research has aimed to discover mechanisms that might protect the myocardium from ischemic damage (Pyo et al., 2008). Since cardiomyocytes have limited regenerative capacity, modulating their function to prevent oxygen deprivation is a promising way to decrease mortality from CVD. There are several mechanisms involved in the development of CVDs. Mitochondrial deregulation, particularly in cardiomyocytes, is intensely involved in the development and progression of CVD (Fan et al., 2020). In pathological states such as ischemia or hypoxia, many genes and enzymes associated with mitochondria are upregulated, leading to mitochondrial dysfunction in cardiac muscle cells and subsequent myocardial damage (Huang & Zhou, 2023). Moreover, cardiomyocytes and their mitochondria are the essential energy producers and consumers of the heart, and their alteration in metabolite profiles during hypoxia or ischemia seems to be a key driver of pathological processes (Grass et al., 2022). In addition, alternation in epigenetic modifications is closely associated with CVDs. These mechanisms regulate CVD‐related gene expression via DNA methylation, noncoding RNA, or histone modification, thus affecting CVD progression. Epigenetic markers represent crucial molecular markers of CVD because they occur in the early stage of the disease and involve key cardiovascular pathologically associated pathways (Shi et al., 2022). Empagliflozin (EMPA) is a sodium‐glucose cotransporter 2 (SGLT2) inhibitor used as an oral medication for treating and managing type 2 diabetes mellitus (T2DM) by improving glycaemic control, reducing glucotoxicity, enhancing glucose metabolism, and decreasing insulin resistance. Furthermore, there is evidence suggesting that EMPA reduces the risk of cardiovascular‐related death and hospitalizations due to heart failure in both patients with T2DM and those without it (Fitchett et al., 2018; Liang & Gu, 2022). EMPA demonstrates beneficial effects, and its pharmacological impact, underlying its cardiovascular benefits, is not entirely understood. It is evident that the pleiotropic role includes significant effects beyond blood glucose reduction (Rastogi & Januzzi, 2023).

Based on the knowledge mentioned above, the main aim of our study was to evaluate the impact of EMPA on human primary cardiomyocytes isolated from human heart ventricles under hypoxic conditions, using cobalt (CoCl_2_) as a model of chemically induced hypoxia. The specific aim of the study is to reveal up‐to‐date unidentified effects of EMPA treatment used in clinical practice. The focus of the study is put on the possible prevention of mitochondrial dysfunction, changes in cell viability, miRNA expression, and metabolomic modulation, which contribute to the initiation and development of CVDs.

## 2 MATERIALS AND METHODS

### 2.1 Cellular model

For the experimental evaluation of the effect of EMPA (MedChemExpress, Princeton, USA, cat. no. HY‐15409), Human Cardiac Myocytes (Merck, Darmstadt, Germany, cat. no. C‐12810) were obtained. The cells were incubated at 37°C in an atmosphere of 5% CO_2_. The cultivation of cardiomyocytes was performed using a medium supplemented according to the manufacturer's instructions. The effect of EMPA was assessed after exposure of the cell culture to a final concentration of 0.5 μM EMPA for 24 and 48 h. The concentration was selected according to previous studies, where the higher (25 mg) recommended clinical dose of EMPA (Sizar et al., 2025) resulted in a peak plasma concentration of the tested individual identified as a 500 ng/mL (Seman et al., 2013). Additionally, the effect of EMPA was examined under pathological conditions simulating hypoxia. In our study, chemical hypoxia was induced using CoCl₂ at a final concentration of 200 μM for 24 and 48 h.

### 2.2 Imunocytochemistry

For the purpose of immunodetection of HIF‐1A, cultured cardiomyocytes were fixed by 4% paraformaldehyde. After 1 h of blocking by 2.5% goat serum in 0.3% triton X‐100 in PBS, anti HIF‐1A primary antibodies (1:200, cat. SC71247, Santa Cruz Biotechnology, USA) were added to the sample overnight. Anti mouse secondary antibodies conjugated with Alexa Fluor‐594 (1:2000, cat. A‐11032, Thermofisher Scientific, USA) together with DAPI counterstaining were added for 1 h. Samples were washed during the protocol between each step by 0.3% Triton X‐100 in PBS for 5 min. Images were obtained by Zeiss LSM 880 at a total of 400× magnification with an additional 3× optical zoom.

### 2.3 Visualization of the mitochondrial network via confocal microscopy

**FIGURE 1:**
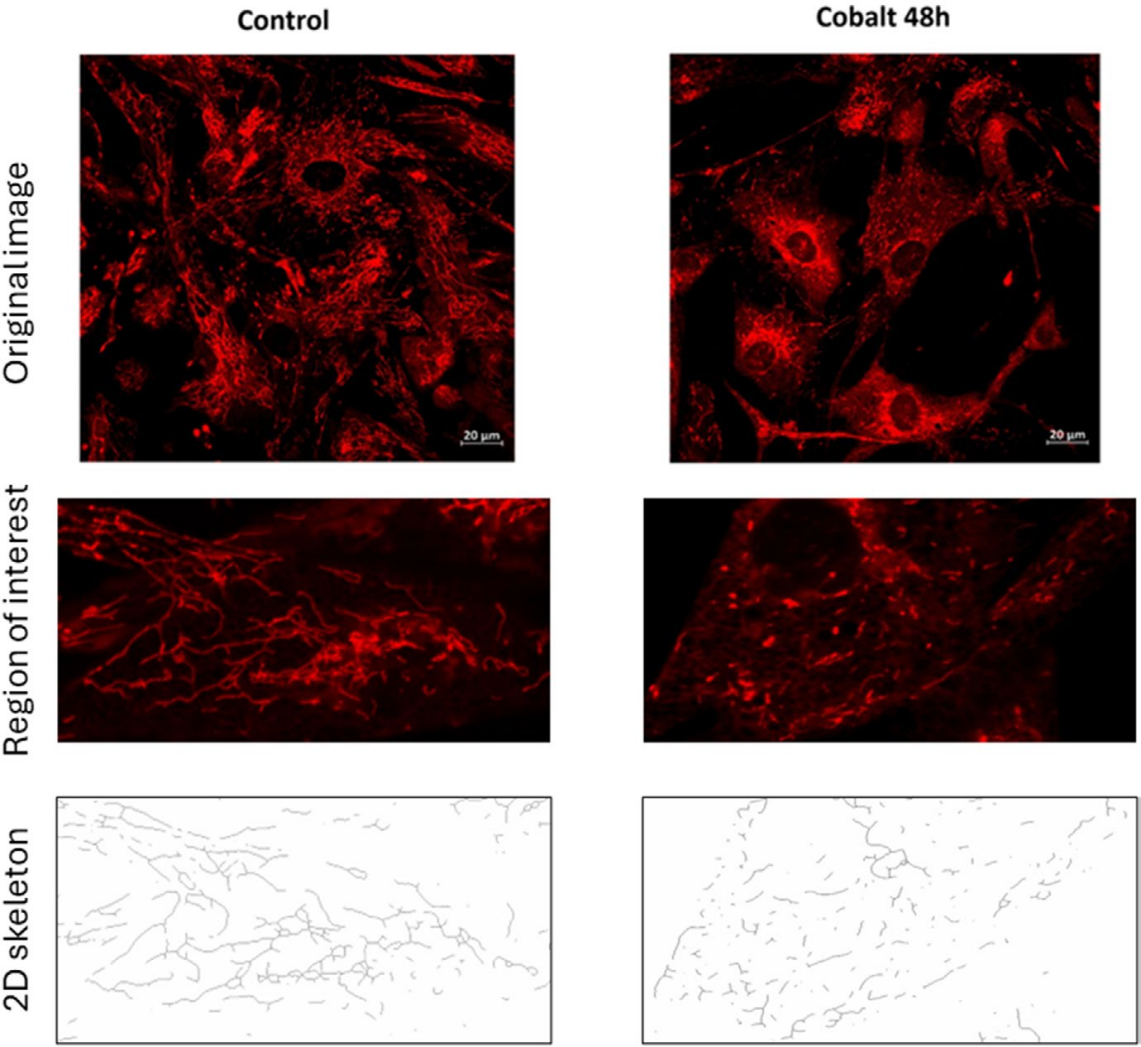
Representative images of the mitochondrial network and their post processing 2D outcome used for the purpose of parametrization of mitochondrial branches. Schema illustrating specific grades (three lines) of continuous processing of representative images obtained from the control and cobalt‐treated cardiomyocyte culture.

To fluorescently label the mitochondrial network in the live cell culture, cardiomyocytes were incubated for 45 min with the Mitotracker RedFM probe (Thermo Fisher Scientific, Waltham, MA, USA) following the manufacturer's instructions. The fluorescent probe was subsequently washed out with fresh medium, and the mitochondrial network was visualized at a total magnification of 400× using a Zeiss LSM 880 scanning confocal microscope (LSM AxioExaminer platform) equipped with a W Plan‐Apochromat 40×/1.0 DIC M27 water‐immersion objective. LSM 880 is operated by the ZEN black software system developed by Zeiss. Mitochondrial network images were post‐processed into 2D skeletons and analyzed using the MiNA plug‐in in ImageJ (FIJI) software, following the methodology of Evinová et al., 2024. The analysis included mitochondrial network branching (number of branching points per mitochondrial branch), mitochondrial fragmentation (number of isolated mitochondria per total mitochondrial branches), and the average mitochondrial branch length within the analyzed cells. Graphical illustration of the protocol as well as representative images of the mitochondrial network are illustrated by Figure 1. The experiment performed in standard/control conditions included seven biological replicates for 24‐h EMPA exposure and eight for 48‐h exposure. The initial number of cells used for experiments performed on 30 mm dishes was 0.2 × 10^6^ cells. To assess EMPA effects under both control and pathological conditions, experiments were conducted with three biological replicates for 24‐h exposure and four for 48‐h exposure.

### 2.4 Cell count

In addition to mitochondrial network visualization, imaged cell cultures were used to assess average cell count and its variations across experimental groups and exposure durations. Cell counting was performed in approximately five randomly selected fields of view per experimental group in three biological replicates.

### 2.5 ^1^H‐NMR spectra acquisition and analysis

The stock solution consisted of a 500 mM phosphate buffer (pH 7.4, measured using a pH meter) with approximately 0.2 mM TMSP‐d₄ (trimethylsilylpropionic acid‐d₄) (Merck, Darmstadt, Germany cat. 269913‐1G) as a chemical shift reference, prepared in deuterated water. A total of 500 μL of centrifuged cell media was mixed with 100 μL of the stock solution and carefully transferred into a 5 mm NMR tube.

NMR spectra were acquired using a 600 MHz Bruker Avance III NMR spectrometer equipped with a cryoprobe at a temperature of 310 K. The evaluation followed a modified Bruker CPMG profiling protocol, with an FID size of 64 k, four dummy scans, 128 scans in total, a spectral width of 20.0156 ppm, and a relaxation delay of 4 s. Peak multiplicities were confirmed using J‐resolved spectra, while homonuclear cross‐peaks were verified in COSY spectra (Table 1). The calculated peak integrals were used as relative concentrations of metabolites in the cell medium collected after 24‐h and 48‐h treatments with cobalt and EMPA, as described above. The initial number of cells used for the presented experiments was set at 0.8 × 10^6^ cells cultivated in a T25 flask. The obtained values were normalized to the cell count in the corresponding experimental groups, measured in three biological replicates for both the 24‐h and 48‐h treatments.

**TABLE 1:** Chemical shifts (in ppm), J couplings (in Hz) and multiplicities (s, singlet; d, doublet; q, quartet; m, multiplet) for the pool of metabolites identified in cell medium by NMR.

Metabolite	NMR peak assignment, confirmed using J‐resolved and cosy spectral analyses
Glutamine	2.1167 (m), 2.1534 (m), 2.441 (m), 2.477 (m), 3.766 (dd)
Histidine	7.067 (s), 7.796 (s)
Isoleucine	0.941 (t; J = 7.48), 1.0123 (d; J = 6.99), 3.678 (d; J = 4.17)
Leucine	0.958 (d; J = 6.23), 0.969d (d; J = 6.05), 1.679 (m), 1.720 (m), 1.749 (m)
Phenylalanine	3.126 (m), 3.284 (m), 7.3403 (d; J = 7.46), 7.383 (t; J = 7.36), 7.436 (t)
Threonine	1.329 (d), 3.582 (d; J = 4.93), 4.253 (m)
Tryptophan	7.2087 (t), 7.292 (t), 7.334 (s), 7.556 (d), 7.743 (d; J = 8.02)
Valine	0.9936 (d; J = 7.06), 1.044 (d; J = 7.06), 2.273 (m), 3.607 (d; J = 4.40)
Pyruvate	2.376 (s)
Glucose	3.233 (m), 3.398 (m), 3.458 (m), 3.524 (dd), 3.782 (m), 3.824 (m), 3.889 (dd), 4.634 (d), 5.233 (d)
Glutamate	2.064 (m), 2.130 (m), 2.357 (m), 3.766 (dd)

### 2.6 Analysis of miRNA expression

Cell culture used for this type of analysis was prepared on 24 well plates by initial seeding of 10,000 cells per well. Total RNA from the cells was isolated using a commercial miRVana miRNA Isolation Kit (Thermo Fisher Scientific, Waltham, MA, USA, cat. AM1560), following the manufacturer's instructions step‐by‐step. The obtained RNA concentration was measured with a NanoDrop ND‐2000 spectrophotometer (Thermo Fisher Scientific, Waltham, MA, USA). For RT‐qPCR analysis, the minimum required concentration of input RNA was 10 ng/μL. Isolated RNA was stored at −80°C in a refrigerator until further processing. Reverse transcription of RNA was conducted using the TaqMan Advanced miRNA cDNA Synthesis Kit (Applied Biosystems, Waltham, MA, USA, cat. A28007). Quantitative real‐time PCR was performed with miRNA‐specific TaqMan™ advanced miRNA assay kits (Applied Biosystems, cat. A25576) for hypoxia‐associated miR‐210‐5p. MiR‐191‐5p was selected as an internal control for normalization in miRNA quantification. Quantitative real‐time PCR was performed on the FX96 real‐time PCR detection system (Bio‐Rad Laboratories, Hercules, CA, USA) in a 10‐μL reaction volume. We conducted three rounds of experiments to ensure the reproducibility of our study. Each biological replicate included three technical replicates of the PCR reaction. Fold changes were calculated following the standard ΔΔCq method.

### 2.7 Statistical analysis

Statistical comparisons of the results obtained from the analyses within individual groups were performed using factorial ANOVA, with EMPA as a single variable factor under control conditions. In experiments conducted under both control and pathological conditions, the statistical evaluation considered the effects of two factors: EMPA and cobalt. A *p* value of ≤0.05 was deemed statistically significant. All statistical analyses were performed in GraphPad Prism 8.1.1 (GraphPad, San Diego, CA, USA).

The degree of significance for the factors is indicated above the graphs as follows: *p* < 0.05, *p* < 0.01, and *p* < 0.001.

## 3 RESULTS

The effect of EMPA on intracellular changes in cardiomyocytes was analyzed under both simulated control and pathological conditions in vitro. The drug's impact on the selected cell type was systematically assessed through mitochondrial network alterations, cell count analysis, changes in metabolic energy‐related metabolites, amino acid consumption from the medium, and variations in selected miRNA.

### 3.1 EMPA effects on mitochondrial morphology

**FIGURE 2:**
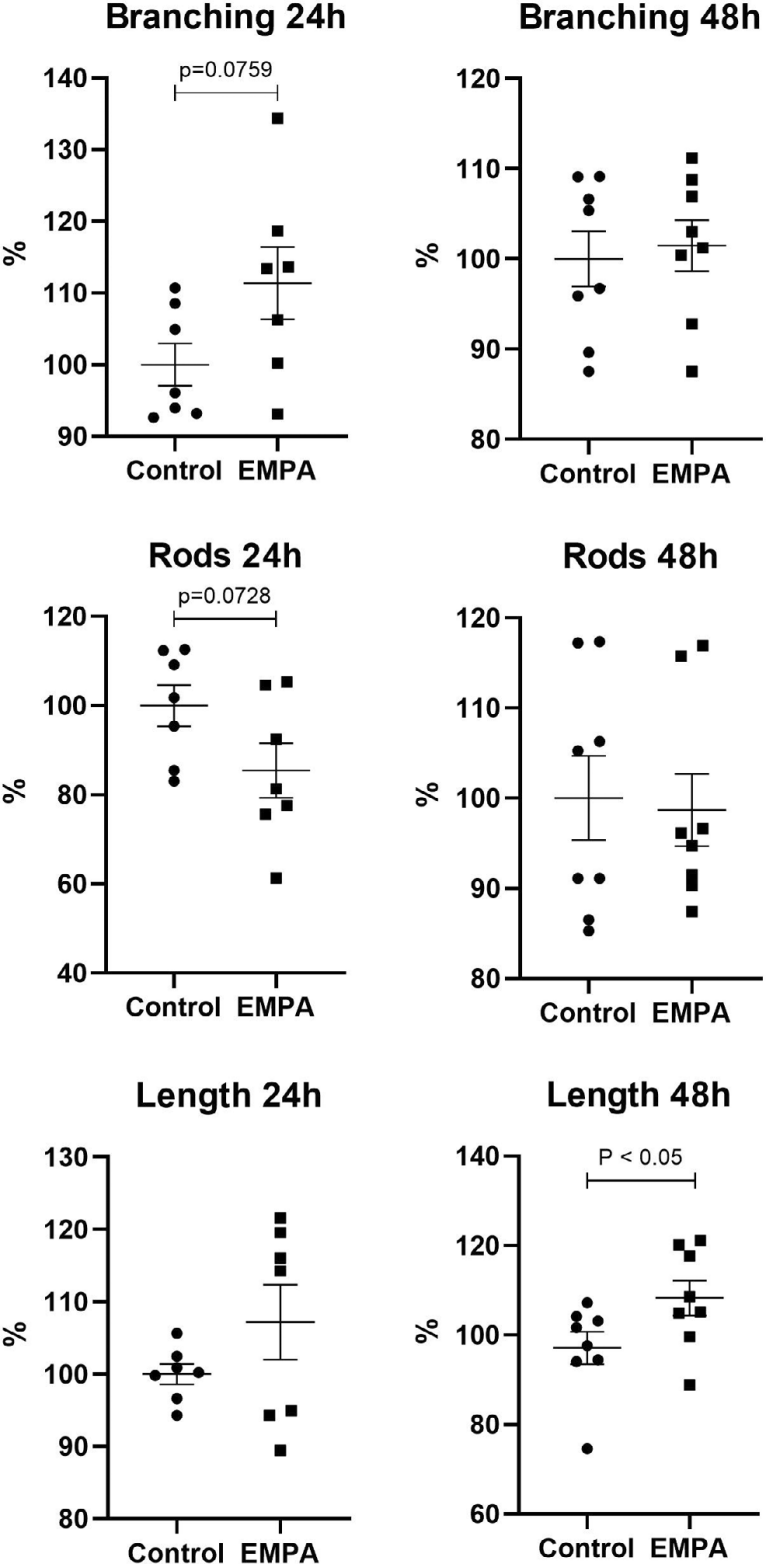
Modulatory effect of EMPA on mitochondrial morphology. Graphical visualization comparing mitochondrial branching, solitary mitochondria count, and average mitochondrial length between controls and cells treated by EMPA during 24 and 48 h.

The beneficial effects of EMPA were observed in the modulation of mitochondrial network structure. After 24 h of exposure, a comparison across seven experimental replicates revealed that EMPA enhances mitochondrial network complexity, as reflected in an increased branching pattern (Figure 2). Additionally, a decreased tendency for mitochondrial fragmentation was identified, which can be linked to improved mitochondrial function (Figure 2). However, both effects diminished after prolonged exposure to EMPA for 48 h. Instead, after 48 h, a borderline significant tendency toward mitochondrial elongation was detected in the cell culture exposed to EMPA compared to control cells (Figure 2).

### 3.2 EMPA effects on mitochondrial morphology under hypoxic conditions

**FIGURE 3:**
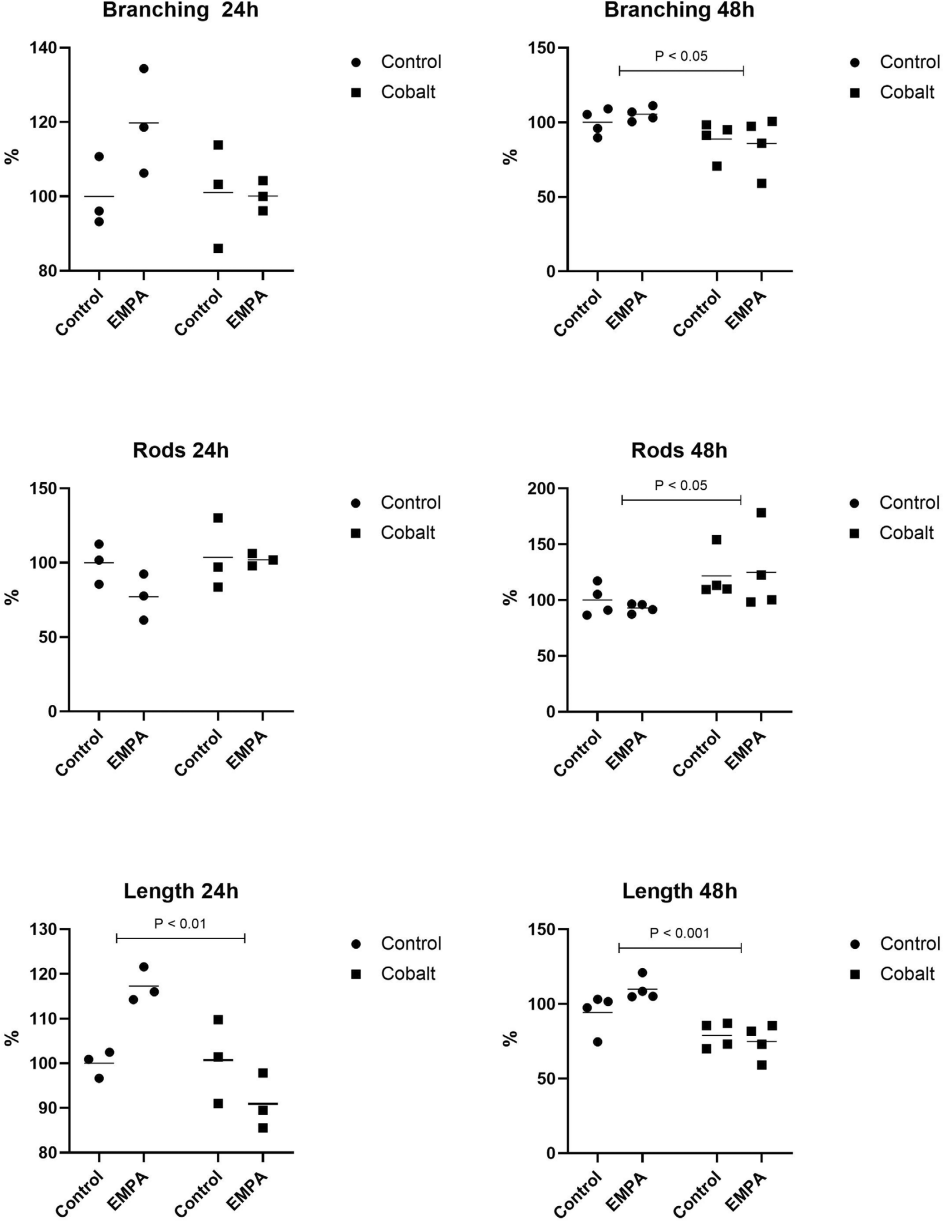
Modulatory impact of EMPA on mitochondrial morphology in cobalt‐mediated hypoxia. Graphical visualization comparing mitochondrial branching, solitary mitochondria count, and average mitochondrial length between cells cultivated with or without EMPA in normoxic and hypoxic conditions during 24 and 48 h.

Another aspect of EMPA's effects was assessed under pathological hypoxic conditions, which were induced by exposure to cobalt chloride for 24 and 48 h. The toxic effect of cobalt ions was particularly evident after 48 h, as it led to a reduction in mitochondrial network branching and, conversely, an increase in mitochondrial fragmentation (Figure 3). The dysfunctional mitochondrial network was further documented by a decrease in the average mitochondrial length, observed not only after 48 h but also as early as 24 h following cobalt exposure (Figure 3).

Despite its effects observed under control conditions, EMPA did not exhibit sufficient robustness to counteract or mitigate the detrimental impact of cobalt ions. This is most clearly reflected in the mitochondrial branch length during 24‐h exposure to both factors, where the statistically significant positive effect of EMPA seen in control conditions was absent in cell cultures exposed to both EMPA and cobalt for 24 h.

### 3.3 Cell count analysis

**FIGURE 4:**
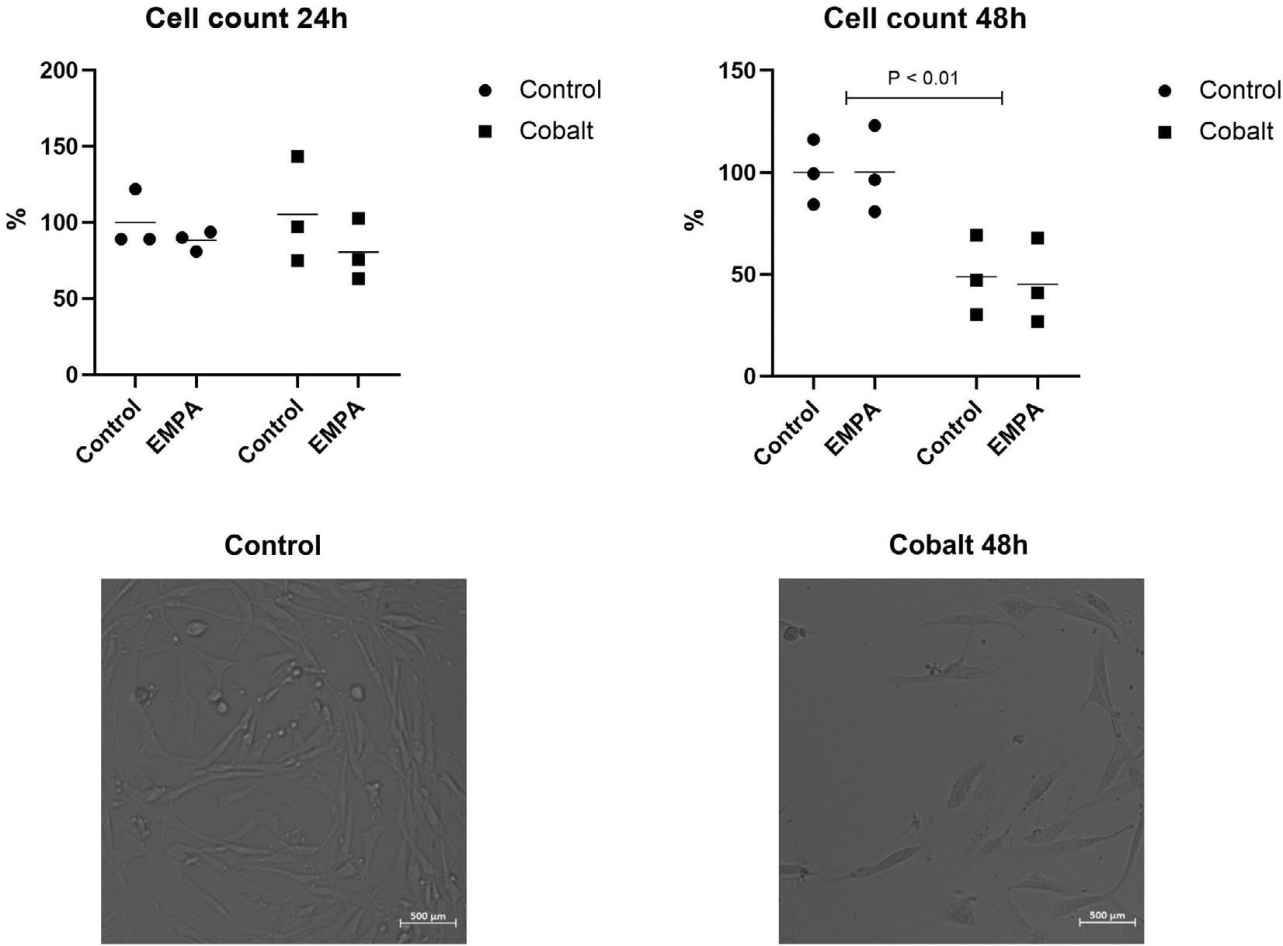
Impact of used treatment in time on cell count. Graphical representation comparing the number of cells after treatment by EMPA in physiological and hypoxic conditions mimicked by cobalt. Lower line provides representative images of control and 48 h cobalt‐treated cardiomyocyte culture acquired in bright field.

In addition to structural mitochondrial alterations, cobalt toxicity also impacted cell count. While no statistically significant reduction was observed after 24 h, a marked decrease in cell count was evident after 48 h in both groups (Cobalt and Cobalt+EMPA) exposed to this substance (Figure 4). EMPA did not influence cell count, neither under control conditions nor under pathological conditions.

### 3.4 Analysis of metabolite levels

**FIGURE 5:**
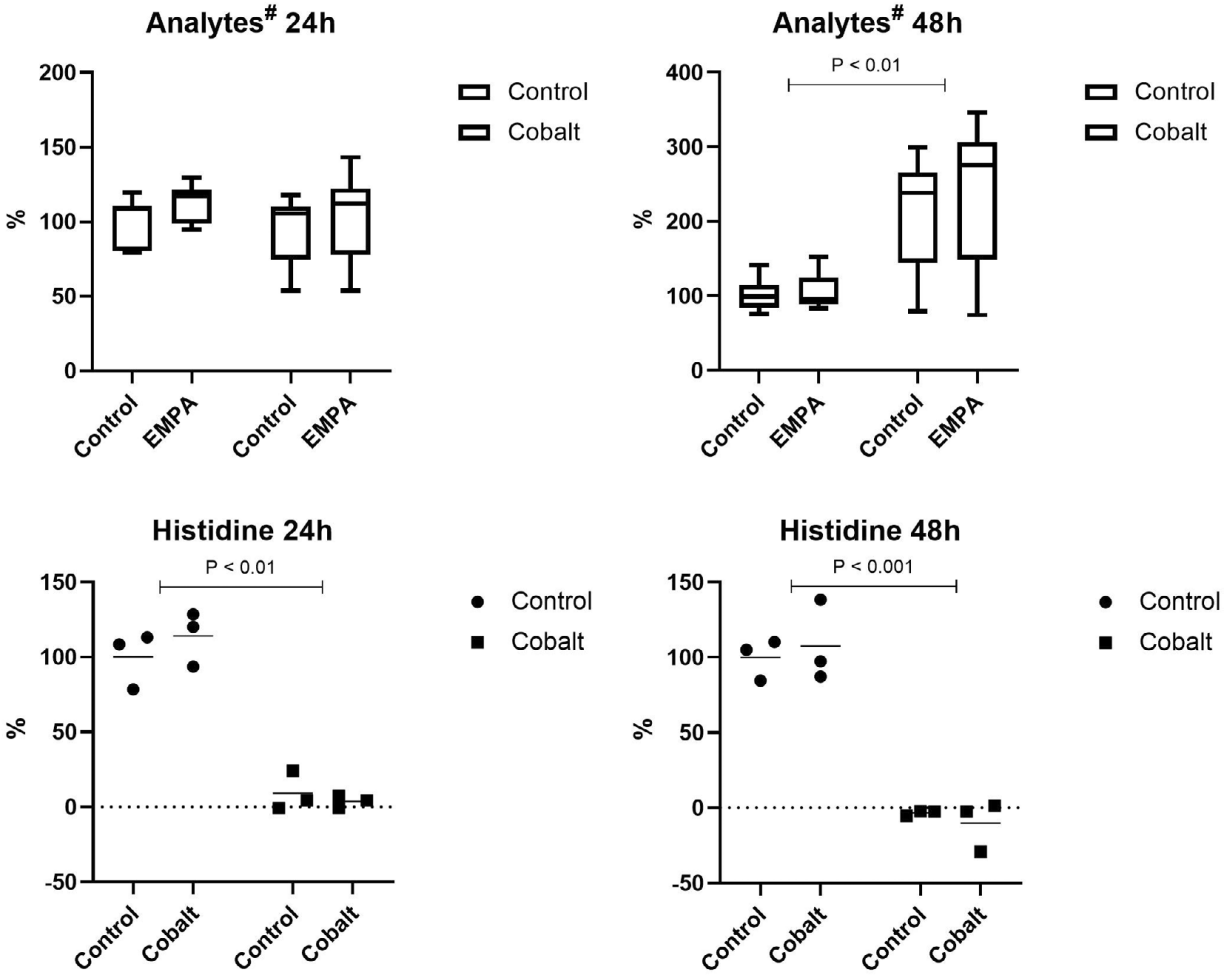
The effect of EMPA on metabolomic profiling. Comparison of amino acids (BCAA, tryptophan, glutamine, glutamate, phenylalanine, and threonine) and energy metabolites (glucose and pyruvate) levels in cell culture medium following treatment with EMPA and cobalt versus control (top panel). The treatment had a similar effect on all of these metabolites, which are therefore presented in a single graph. The bottom panel shows histidine levels, where an opposite and significantly more pronounced effect was observed compared to the other amino acids and metabolites. # Analytes represent the average levels of all amino acids (except histidine) and energy metabolites under the tested conditions. Individual graphs of separate metabolites are provided as supplementary data.

The impact of cobalt ions was further reflected in increased concentrations of nearly all analyzed metabolites in the culture media surrounding the cells (Figure 5). Using nuclear magnetic resonance (NMR), a significant increase in the concentration of amino acids (BCAA, tryptophan, glutamine, glutamate, phenylalanine, and threonine) and energy metabolites (glucose and pyruvate) was detected after 48 h of cobalt exposure. An intriguing observation was the pronounced reduction in histidine levels at both time points following cobalt exposure. Unlike cobalt, EMPA did not exhibit any significant effect on the measured amino acid or energy metabolite profile as documented in Figure 5, representing the average metabolite concentrations in the medium. All measurements of individual analytes are summarized in the Figure S1.

### 3.5 The modulatory effect of hypoxia mimicking conditions on miRNA and HIF1A expression

**FIGURE 6:**
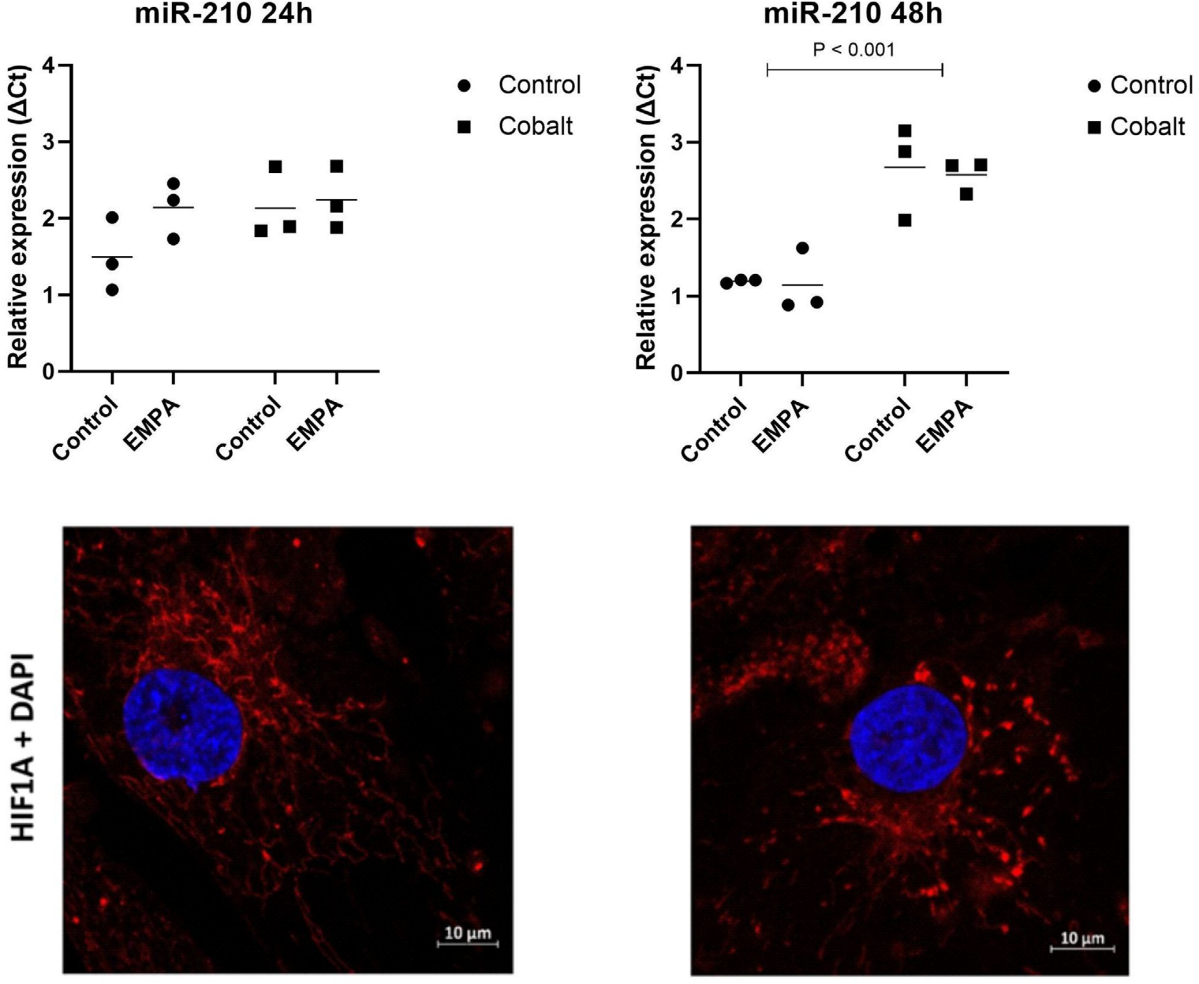
Impact of cobalt treatment on factors associated with intracellular hypoxic conditions. Graphical illustration of relative change of expression of microRNA 210 after 24 h (left) and 48 h (right) of simultaneous and separate treatment by cobalt ions (CoCl_2_ 200 μM) and EMPA (empagliflozin 0.5 μM) compared to control cells. The bottom line shows results of immunodetection of HIF‐1α in control and after 48 h of cobalt presence in growth medium. DAPI staining of nuclei was used as a counterstain.

No significant changes in miR‐210‐3p expression were observed in cells treated with EMPA under control conditions for either 24 or 48 h. Likewise, expression levels remained unchanged in cells exposed to cobalt for 24 h. However, prolonged exposure to cobalt for 48 h resulted in a statistically significant increase in miR‐210‐3p expression, with a fold change of 2.94 in untreated cells and 2.63 in the cells treated with EMPA. The significance of the cobalt effect was confirmed also by the change of quality pattern of HIF1A intracellular presence. Diffuse signal associated with the supposed mitochondrial network is replaced by higher density HIF1A points related to slowed degradation of hypoxic factor after the introduction of cobalt into cell culture for 48 h. Expression of miR‐210‐3p and HIF1A in treated cells under control and hypoxic conditions is presented in Figure 6.

## 4 DISCUSSION

Myocardial ischemia is a pathological condition characterized by reduced blood flow and oxygen supply (hypoxia). This state is strongly associated with cardiomyocyte dysfunction, leading to their apoptosis or necrosis and subsequent development of myocardial ischemia injury (Shen et al., 2024). There are several mechanisms by which hypoxia can promote cardiomyocyte apoptosis on mitochondrial, metabolomic, or epigenetic levels (Garbern & Lee, 2021; Gilsbach et al., 2018). The current research focused on CVD is intensively studying mechanisms to protect cardiomyocytes from hypoxia‐induced injury (Yu et al., 2022). EMPA exerts significant benefits for patients with CVD connected to a reduced rate of cardiovascular mortality, but its direct mechanisms of action are not fully understood. In our study, we assessed the cardioprotective effect of the SGLT2 inhibitor EMPA on human primary cardiomyocytes in the chemically induced model of hypoxia using CoCl_2_. For these purposes, we conducted experiments to assess the potential pleiotropic effect of EMPA by (1) evaluating differences in mitochondrial network integrity, (2) determining cell count, (3) quantifying alterations in metabolomic profiling, and (4) determining alterations in miRNA expression.

The mitochondrial network is defined as a good indicator of cell health and plays a crucial role in maintaining cell function and integrity. Recent evidence suggests that the integrity of the mitochondrial network in cardiomyocytes from failing hearts is impaired due to hypoxia. Ions such as Co^2+^ can mimic hypoxic phenotype and stabilize HIF‐1α in normoxic conditions. Evidence shows that HIF‐1α attenuates biogenesis and oxidative phosphorylation and accelerates mitophagy (Packer, 2021). In our study, EMPA improved mitochondrial network complexity in cardiomyocytes through the increase in branching (*p* < 0.05) under physiological (normoxic) conditions compared to controls. These findings confirm the modulatory role of gliflozins connected to mitochondrial structure and function (Belosludtsev et al., 2021; Zügner et al., 2022). On the other hand, no protective effect of EMPA on mitochondrial morphology was documented in the presence of CoCl_2_. Our data indicate that although EMPA can improve mitochondrial network integrity, using Co^2+^ as a hypoxia‐mimicking agent negates these beneficial effects.

Hypoxia is commonly understood as physiological stress, in which cells facing oxygen deprivation initially utilize adaptive and survival strategies. If hypoxia is sustained, cell death will ultimately occur (Azad et al., 2008). In the study by Chang et al., HK2 cells pretreated with dapagliflozin significantly enhanced cell viability under hypoxic conditions induced by ischaemia in a dose‐dependent manner (Chang et al., 2016). Our data showed a significant decrease in cell count after cobalt intervention. Subsequently, EMPA treatment couldn't prevent the activation of signaling pathways leading to cell death. Although the different used model for hypoxia simulation, in future experiments, the preconditioning effect of empagliflozin in the cobalt‐based model should be tested.

Epigenetic regulation, including miRNA expression, plays a crucial role in the occurrence and development of CVD (Shi et al., 2022). Small noncoding miR‐210 is significantly upregulated during hypoxia, and it can serve as a potential biomarker of myocardial ischemia. Recent studies confirmed that miR‐210 specifically is the robust target of the HIF‐1 transcription factor. Furthermore, HIF‐1 induction of miR‐210 also stabilizes HIF‐1A through a positive regulatory loop (Grosso et al., 2013), what our results are also in favour with (Figure 6). Overall, increased levels of miR‐210 are cardioprotective, connected with cardiomyocyte proliferation and suppressing apoptosis (Bei et al., 2024). EMPA, as a pleiotropic drug, showed a regulatory effect on epigenetic modification in the study by Steven et al. (2017). Our study identified a decreasing tendency (but not significant) of miR‐210 expression after EMPA intervention in cells cultivated with cobalt. This decline may indicate a reduction in HIF‐1 activity and, consequently, alleviate the hypoxic phenotype after EMPA treatment.

Our last analysis was focused on assessing the metabolomic changes in the culture media surrounding the cells. Cobalt significantly increased the quantity of amino acids and energy metabolites in the cell‐cultivated medium regardless of EMPA treatment. Increased secretion of amino acids into cell culture medium could result from the cell response to stress and altered metabolism as a part of the adaptation process connected to changes in transport mechanisms that regulate amino acid uptake and efflux (Harned et al., 2014; Wang et al., 2018). Higher concentrations of extracellular amino acids may indicate their release from cell culture after the onset of cell apoptosis caused by cobalt exposure. However, the most probable mechanism resulting in an increase in metabolite concentration in the growth medium could be their reduced consumption by the depressed level of metabolism in general, which is a well‐known impact of cobalt ions (Díez‐Tercero et al., 2021). On the other hand, the histidine level was significantly decreased in the growth medium of cells cultivated with cobalt compared to control cells. A significant shift in histidine is likely caused by the formation of cobalt‐histidine complexes, which altered the NMR spectra of the metabolite (Morris & Martin, 1970). However, it is necessary not to forget that histidine could serve as a precursor of histamine. This type of amine is crucial for sustaining cell survival as an agent that reduces the extent of damage during oxygen deprivation in the myocardium (Zhang et al., 2018). In our study, we identified a significant decrease in histidine in the cell culture medium, indicating elevated histamine production due to mimicking hypoxic conditions. Subsequent intervention using EMPA did not affect this phenomenon. The hypothesis needs to be verified by an independent measurement of histidine/histamine levels using an additional method.

## 5 CONCLUSION

In conclusion, we analyzed the potential cardioprotective effect of EMPA in cardiomyocytes under normoxic and hypoxic conditions using cobalt as a hypoxia‐inducing agent. The application of EMPA improved mitochondrial morphology and was associated with the integrity of mitochondrial networks. These findings suggest the potential benefits of EMPA in preventing ischemic injury in CVD. Although we observed some cardioprotective effects of EMPA, the results were not sufficiently robust; further investigation of additional cellular‐level parameters of myocardial ischemia may be necessary to clarify the pleiotropic effects of EMPA in CVD.

## FUNDING INFORMATION

Experiments were funded by the VEGA 1/0090/20 grant of the Scientific Grant Agency of the Ministry of Education, Science and Sports of the Slovak Republic.

## CONFLICT OF INTEREST STATEMENT

No potential conflict of interest was reported by the author(s).

## ETHICS STATEMENT

Not applicable.

## Supporting information


Figure S1.

## Data Availability

On reasonable request from the corresponding author.
