# Supplementary figures and images for: Effect of empagliflozin on human primary cardiomyocytes in a chemically induced hypoxia by CoCl2
Source: Physiol Rep. 2025 Dec 10;13(23):e70653. doi: 10.14814/phy2.70653 (PMC12696029; doi:10.14814/phy2.70653)

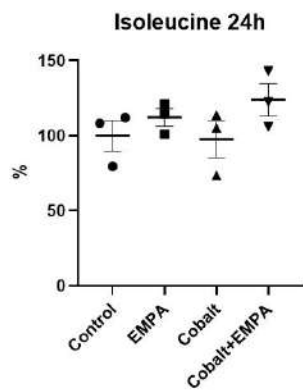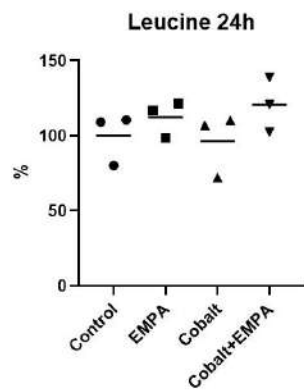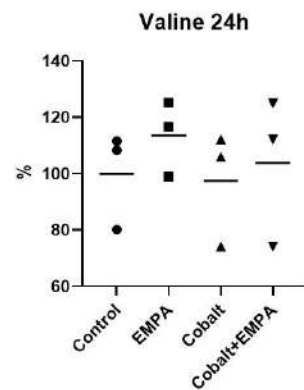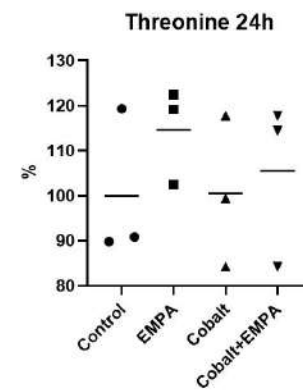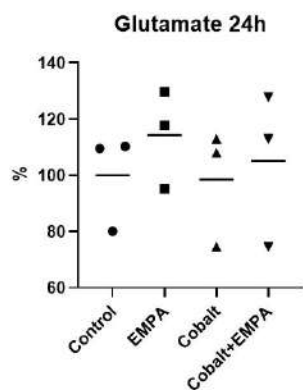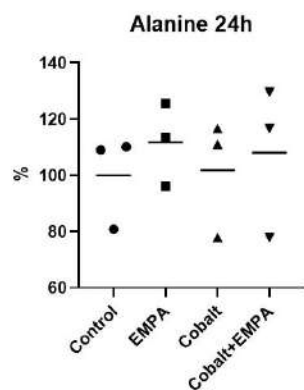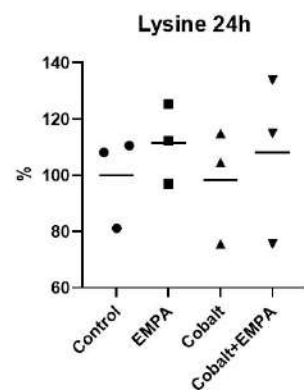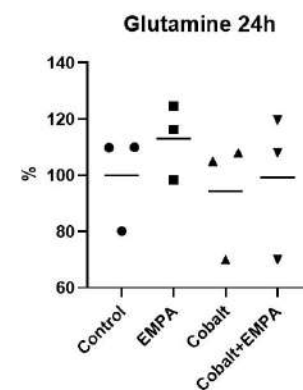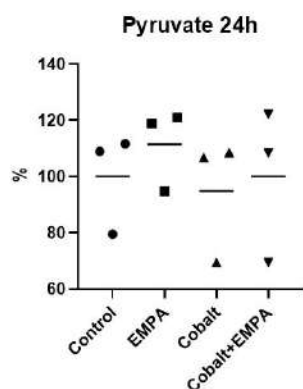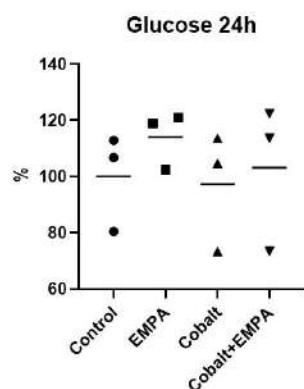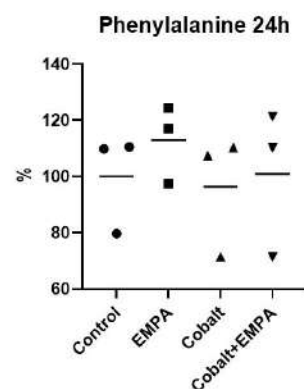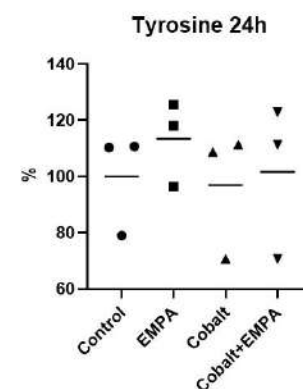

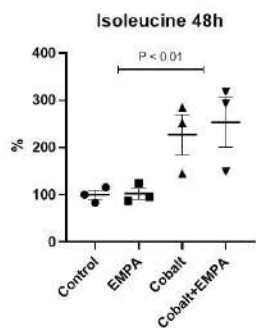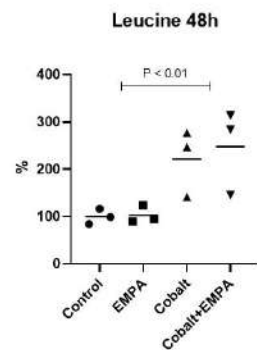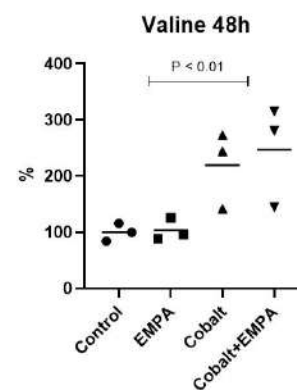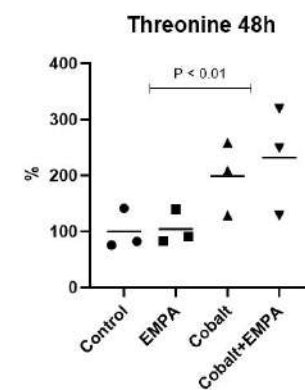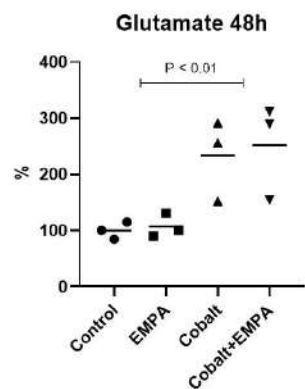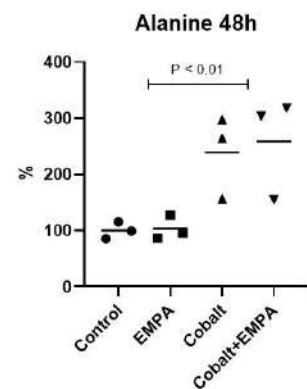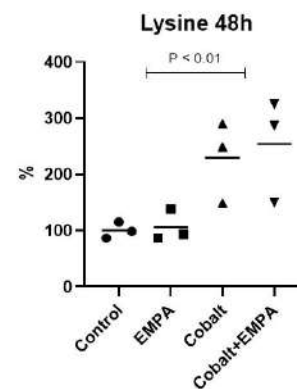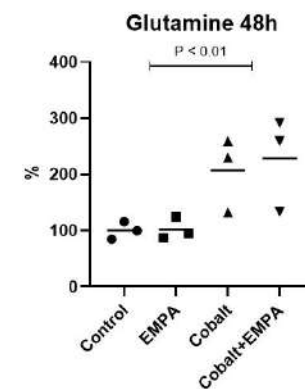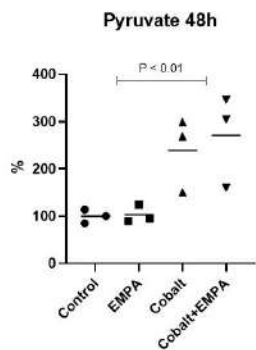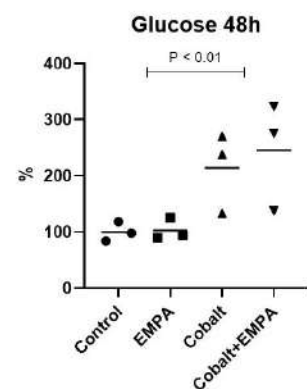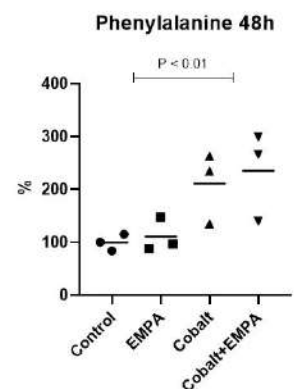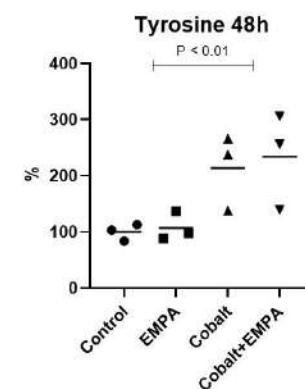

Supplement: Supplementary file 1 — Figure S1. [file PHY2-13-e70653-s001.pdf]
